# Neutrophil extracellular trap (NET)-related index as an indicator of periprosthetic joint infection

**DOI:** 10.5194/jbji-11-363-2026

**Published:** 2026-06-22

**Authors:** Tingrun Cui, Yongjian Liang, Zeyu Feng, Libo Hao, Guoqiang Zhang, Ming Ni, Jing Sheng, Dihua Shangguan, Jiying Chen, Jun Fu

**Affiliations:** 1 Department of Orthopaedics and Traumatology, Beijing Jishuitan Hospital, Capital Medical University, Beijing, 100035, China; 2 National Center for Orthopaedics, Beijing, 100035, China; 3 Department of Orthopaedics, the First Medical Center of Chinese PLA General Hospital, Beijing, 100053, China; 4 Senior Department of Orthopaedics, the Fourth Medical Center of Chinese PLA General Hospital, Beijing, 100048, China; 5 Beijing National Laboratory for Molecular Sciences, Key Laboratory of Analytical Chemistry for Living Bio-systems, CAS Research/Education Center for Excellence in Molecular Sciences, Institute of Chemistry, Chinese Academy of Sciences, Beijing, 100190, China; 6 School of Chemical Sciences, University of Chinese Academy of Sciences, Beijing, 100049, China

## Abstract

**Background**: Periprosthetic joint infection (PJI) is a devastating complication of arthroplasty and is difficult to diagnose accurately. This study aims to explore the value of neutrophil extracellular traps in synovial fluid (SF-NETs, web-like structures released by neutrophils as a critical innate immune response) for diagnosing PJI. **Methods**: A retrospective cohort study was conducted, enrolling post-arthroplasty subjects from January 2018 to December 2023. Three components of SF-NETs (cell-free double-strand DNA, SF-dsDNA; citrullinated histone H3, SF-CitH3; SF-Nucleosome) and SF-NETs_1+_ (positive with one out of the three NET components), SF-NETs_2+_ (positive with two out of the three NET components), white blood cell count (SF-WBC), polymorphonuclear cell percentage (SF-PMN %), neutrophil count in SF (SF-PMN), microbiological examinations (Culture) and infection-related systemic indices were evaluated. **Results**: A total of 64 of 153 included subjects were diagnosed as PJI. SF-dsDNA and SF-CitH3 had significantly higher levels in the PJI group compared to the non-PJI group ( P < 0.001) and showed positive correlations with SF-WBC, SF-PMN % and SF-PMN (0.4 < ρ < 0.7), while SF-Nucleosome had no significant difference. Sensitivity and specificity of SF-NETs_1+_, SF-NETs_2+_ and Culture were 82.2 % and 78.7 %, 59.4 % and 95.5 %, and 54.1 % and 86.8 %, respectively. The NET related index (NETRI) was defined as 10.529 × SF-NETs ${}_{1+}+$ 28.114 × SF-PMN % + 8.210 × Culture-1.452, with the area under the receiver operating characteristic curve of 0.922, making it a novel indicator. **Conclusions**: SF-NETs_1+_ and SF-NETs_2+_ may serve respectively in the screening and exclusion of PJI. NETRI represented a new discriminator for PJI diagnosis.

## 1 Introduction

Periprosthetic joint infection (PJI) refers to infection around the joint prosthesis after implantation. Its incidence is about 2 %–4 % and has risen in recent years (Parvizi et al., 2011; Cai et al., 2023). PJI is an unexpected postoperative infection, distinct from primary or latent infections; despite the implementation of multiple evidence-based preventive strategies, PJI cannot be entirely eliminated (Parvizi et al., 2018). Delayed diagnosis and inadequate treatment can cause severe outcomes, prolonged hospitalization, delayed recovery, and heavy psychological and economic burdens (Rao et al., 2011; Parvizi et al., 2018).

Various laboratory markers have been studied for PJI diagnosis, including C-reactive protein (CRP), D-dimer, erythrocyte sedimentation rate (ESR), and synovial fluid (SF) white blood cell (WBC) count (SF-WBC), SF polymorphonuclear cell percentage (SF-PMN %), leukocyte esterase, and microbiological tests, as recommended by guidelines. However, diagnostic uncertainty persists (e.g., “possibly infected” or “infected likely”), and novel markers or techniques have continued to be explored, although many faced practical limitations (McNally et al., 2021; Parvizi and Gehrke, 2014; Parvizi et al., 2018; Sigmund et al., 2022).

Neutrophils are the most abundant effector cells in the innate immune system and play a central role in PJI pathogenesis. Neutrophil-related indices, such as α -defensin, CD-64 and neutrophil extracellular traps (NETs), have attracted attention. While α -defensin is a well-established diagnostic marker and CD64 shows good diagnostic potential, both are limited by time-consuming, costly and clinically inconvenient detection methods (Qin et al., 2020; Yuan et al., 2017). NETs consist of decondensed chromatin (mainly cell-free dsDNA), histones and nucleosomes (consisting exactly of nuclear dsDNA and histones) (Cutter and Hayes, 2015), forming large extracellular structures that trap and kill pathogens (Papayannopoulos, 2018). Recent studies have investigated NET components in synovial fluid (SF) for PJI diagnosis. Lögters et al. (2009) and Cobra et al. (2022) found significant differences in cell-free dsDNA between infection and non-infection groups. Cai et al. (2023) reported an area under the receiver operating characteristic (ROC) curve (AUC) of 0.971 for SF-NETs in 74 cases using a single enzyme-linked immunosorbent assay (ELISA) kit. De Sandes Kimura et al. (2024) showed AUCs of 0.94–1.00 for isolated NET constituents in 32 cases. However, prior studies had relatively small sample sizes and a high risk of bias, leaving changes in individual NET components unclear. We therefore conducted this retrospective cohort study to evaluate the diagnostic value of SF-NET constituents and their combinations for PJI diagnosis. 

## 2 Methods

### 2.1 Subjects and specimens

This retrospective cohort study was conducted in patients with suspected PJI. From January 2018 to December 2023, 172 patients aged 18 years or older with suspected PJI after total joint arthroplasty and who underwent diagnostic laboratory testing were consecutively enrolled in the First Medical Center of Chinese PLA General Hospital. Exclusion criteria were cancer, liver cirrhosis, severe renal insufficiency and the absence of confirmed infection-related diagnoses or necessary data. The diagnosis of PJI was made following the Musculoskeletal Infection Society definition of PJI in 2014 (Parvizi and Gehrke, 2014) integrating all available data under the clinical scenario. This study was approved by the Ethics Committee of Chinese PLA General Hospital. We conducted the study following the principles of the Declaration of Helsinki and current ethics standards. All patients signed the written informed consent.

SF specimens from the included subjects were harvested via aseptic arthrocentesis, which were aliquoted and stored at - 80 °C within 2 h of acquisition. Before storage, routine laboratory tests and pathogen cultures of SF were conducted. When conditions of “punctio sicca” were encountered, joint irrigations with sterile normal saline followed by aspirations of the lavage fluid were performed for microbiological analyses, and cytological studies were thus omitted due to dilution. At the same time, venous blood was collected into three tubes, containing 3.2 % sodium citrate, ethylene diamine tetra-acetic acid (EDTA) and clot activator (BD Biosciences, New Jersey, USA), respectively. Citrated whole blood was centrifuged at 1500 g at room temperature for 15 min to obtain the plasm. Clotted whole blood was centrifuged at 1500 g at room temperature for 15 min to obtain the serum.

### 2.2 Laboratory parameters

WBC count, neutrophil percentage (NEUT %) and platelet (PLT) count in EDTA-anticoagulated whole blood, as well as SF-WBC and SF-PMN %, were determined using a Sysmex XN-20 analyzer (Sysmex, Kobe, Japan). The absolute counts of neutrophils (NEUT) were calculated by multiplying the WBC and NEUT %, and the SF polymorphonuclear cell count (SF-PMN) was calculated as the product of SF-WBC and SF-PMN %. The levels of ESR and CRP in EDTA-anticoagulated whole blood were measured using a Greiner Bio-One SRS 100/II analyzer (Greiner Bio-One, Frickenhausen, Germany) and a Lifotronic PA-990 analyzer (Lifotronic, Shenzhen, China), respectively. The D-dimer level in plasma was measured using a Stago STA R Max analyzer (Stago, Paris, France). The interleukin-6 (IL-6) level in serum was measured using an Immulite 1000 immunoassay system (Siemens, Forchheim, Germany). For pathogen culture, the SF specimens were injected into BACT/ALERT PF Pediatric FAN vials and BACT/ALERT PF FA FAN vials, respectively, and detected using a BioMérieux BACT/ALERT system (BioMérieux, Marcy l'Etoile, France). All specimens for microbiological tests were incubated for up to 14 d unless the positive growth of pathogens, and the results, were documented as “Culture”.

### 2.3 Measurement of SF-NET components

The concentrations of the three main constituents of NETs in SF, including cell-free dsDNA (SF-dsDNA), citrullinated histone H3 (SF-CitH3) and nucleosomes (SF-Nucleosome), were measured (Liu et al., 2021). The SF aliquots were diluted twice with phosphate buffer solution. The kits were read using a SpectraMax M2 microplate reader (Molecular Devices, California, USA).

The Quant-iT™ PicoGreen^®^ dsDNA Assay Kit (Invitrogen, California, USA) was applied to determine the concentration of SF-dsDNA. Two 96-well black plates (Corning, New York, USA) were utilized for fluorescence acquisition, with an excitation wavelength of 480 nm and an emission wavelength of 520 nm. The Citrullinate Histone H3 ELISA Kit (Cayman, Michigan, USA) was employed to measure SF-CitH3, with the absorption wavelength set at 450 nm. The SF-Nucleosome was measured using the Cell Death Detection ELISA^PLUS^ Kit (Roche, Basel, Switzerland), and the value of absorption at the wavelength of 405 nm subtracted from that of 490 nm was adopted. The absorbance value of the negative control well was subtracted from that of the test well and the positive control well, respectively. Subsequently, the ratio of the resulting value for SF-Nucleosome concentration was calculated.

### 2.4 Statistical analysis

An IBM SPSS 26.0.0 (New York, USA) and GraphPad Prism 8.0.2 (Massachusetts, USA) were employed for statistical analysis. All data had non-normal distributions; thus, they were presented as median (25th, 75th percentiles). A Mann–Whitney U test was used to identify variables with statistically significant differences between the PJI and non-PJI groups. Spearman's rank correlation coefficient ρ was used for nonparametric correlation analysis. Outliers twice the interquartile range and larger than the 75th percentile were excluded in the linear regression analysis. Binomial logistic regression analysis was used to generate odds ratios (ORs), with 95 % confidence intervals (CI). An ROC curve analysis was adopted to estimate the diagnostic efficacy of the respective indices. All box plots were presented with the horizontal bars representing medians and the vertical bars representing the 2.5th and the 97.5th percentiles. P < 0.050 was considered as statistically significant. 

## 3 Results

### 3.1 Demographic and clinical characteristics of subjects

Of the total of 172 subjects, 19 fulfilled the exclusion criteria, and the rest were included in the study. Among the 153 subjects, 64 were diagnosed with PJI, all of which were classified as chronic PJI based on the available medical history and diagnostic findings. The demographic and clinical characteristics of the two groups are shown in Table 1. The median age of participants was 67.0 (60.0, 72.0) years, with a significant difference between the PJI and non-PJI groups ( P=0.021 ). There were 41 hips and 112 knees involved in total, with no significant difference between groups ( P=0.494 ).

The results for all parameters in the two groups are displayed in Table 2. We found that PJI patients had higher levels of SF-dsDNA and SF-CitH3 than non-PJI patients ( P < 0.001 for both), but there is no significant difference in SF-Nucleosome ( P=0.135 ).

**Table 1:** Demographic and clinical characteristics of infected and non-infected patients^a^.

Items	PJI group, n=64	Non-PJI group, n=89
Male/female n/n	28/36	39/50
Age, year	69.0 (63.0, 73.5)	66.0 (57.0, 71.0)^b^
Affected joint, hip/knee, n/n	19/45	22/67

^a^ Data proven to be non-normally distributed are showed as median (25th, 75th percentiles). ^b^ P < 0.050, and the difference is statistically significant when compared with patients with PJI. Abbreviations: PJI, periprosthetic joint infection.

**Table 2:** Parameters in infected and non-infected patients^a^.

Parameters	PJI group, n=64	Non-PJI group, n=89
SF-dsDNA, ng mL^−1^	1.84 (0.94, 2.16)	0.23 (0.12, 0.64)^b^
SF-Nucleosome	1.02 (0.49, 2.56)	0.92 (0.47, 1.84)
SF-CitH3, ng mL^−1^	1509.11 (123.33, 3095.45)	24.19 (1.71, 211.60)^b^
SF-WBC, × 10^6^ L^−1^c^^	15 690.00 (2880.00, 26 800.00)	393.00 (160.00, 2001.00)^b^
SF-PMN %^c^	89.00 (73.00, 94.00)	32.00 (14.00, 63.50)^b^
SF-PMN, × 10^6^ L^−1^c^^	12 086.67 (2358.40, 23 450.47)	125.13 (21.04, 933.60)^b^
Culture + , n (%)^d^	33 (54.10)	11 (13.25)^b^
D-Dimer, mg L^−1^ FEU	2.31 (1.25, 3.77)	1.37 (0.60, 2.31)^b^
ESR, mm h^−1^	40.00 (22.50, 69.50)	13.00 (6.00, 21.00)^b^
WBC, × 10^9^ L^−1^	7.11 (5.21, 8.24)	6.06 (5.08, 8.05)
NEUT %	70.35 (61.70, 75.55)	62.30 (54.90, 71.75)^b^
NEUT, × 10^9^ L^−1^	4.74 (3.39, 5.83)	3.71 (2.96, 5.79)
PLT, × 10^9^ L^−1^	240.50 (202.00, 350.50)	219.00 (185.00, 264.50)^b^
CRP, mg dL^−1^	2.19 (0.92, 5.21)	0.22 (0.10, 1.05)^b^
IL-6, pg mL^−1^	14.20 (10.13, 32.90)	5.48 (2.31, 15.28)^b^

^a^ Data proven to be non-normally distributed are showed as median (25th, 75th percentiles). ^b^ P < 0.050, and the difference is statistically significant when compared with patients with PJI. ^c^ Levels of SF-PMN(%) and SF-WBC were examined on SF of 45 randomly selected from 64 PJI patients and 47 from 89 non-PJI patients. ^d^ Culture were evaluated on SF of 61 randomly selected from 64 PJI patients and 83 from 89 non-PJI patients. Abbreviations: PJI, periprosthetic joint infection; SF, synovial fluid; SF-dsDNA, double-stranded DNA in synovial fluid; SF-Nucleosome, nucleosome in synovial fluid; SF-CitH3, citrullinated histone H3 in synovial fluid; SF-WBC, white blood cell in synovial fluid; SF-PMN %, polymorphonuclear cell percentage in synovial fluid; SF-PMN, polymorphonuclear cell count in synovial fluid; ESR, erythrocyte sedimentation rate; WBC, white blood cell; NEUT %, neutrophil percentage; NEUT, neutrophil; PLT, platelet; CRP, C-reactive protein; IL-6, interleukin-6.

Notably, the SF-WBC level in PJI group was almost 40 times higher than that in non-PJI group ( P < 0.001). Compared to the patients without PJI, the levels of SF-PMN % and SF-PMN were higher in those with PJI ( P < 0.001 for both). For Culture, the number of positive cases in the PJI group was significantly more than that of non-PJI group ( P < 0.001); and the sensitivity, specificity, positive predictive value (PPV) and negative predictive value (NPV) were 54.10 %, 86.75 %, 75.00 % and 72.00 %, respectively. Among the 33 Culture-positive subjects in the PJI group, staphylococci were the predominant pathogens, with *Staphylococcus aureus* isolated in eight cases (24 %) and coagulase-negative staphylococci (CoNS) in nine cases (27 %; *S. epidermidis*, *S. haemolyticus*, *S. hominis*, *S. warner*i, *S. caprae* and *S. lugdunensis*). Other organisms included enterococci (four cases), Gram-negative bacilli (four cases), viridans group streptococci and *S. agalactiae* (two cases), *Candida parapsilosis* (two cases), *Brucella* spp. (two cases), and occasional *Micrococcus* and rapidly growing mycobacteria. Three polymicrobial cases were identified. Among the non-PJI cases, 11 had a single positive Culture result, including five CoNS, two Gram-negative bacilli, two *Bacillus* spp. and two *Rhizopus* spp.

There is no significant difference in the level of WBC between two groups ( P=0.293 ). However, compared to non-PJI patients, the level of NEUT % was higher in PJI patients ( P=0.002 ), and the level of NEUT tended to be higher in patients with PJI ( P=0.053 ). Patients without PJI manifested lower levels of D-dimer than those with PJI ( P=0.008 ). Compared to the non-PJI group, the levels of ESR and PLT were higher in patients with PJI ( P < 0.001 and P=0.020 , respectively). Finally, the levels of CRP and IL-6 demonstrated a significant increase in the PJI group compared to the non-PJI group ( P < 0.001 for both). 

### 3.2 Correlation between SF-NET components and WBC-related indices in SF

**Figure 1:**
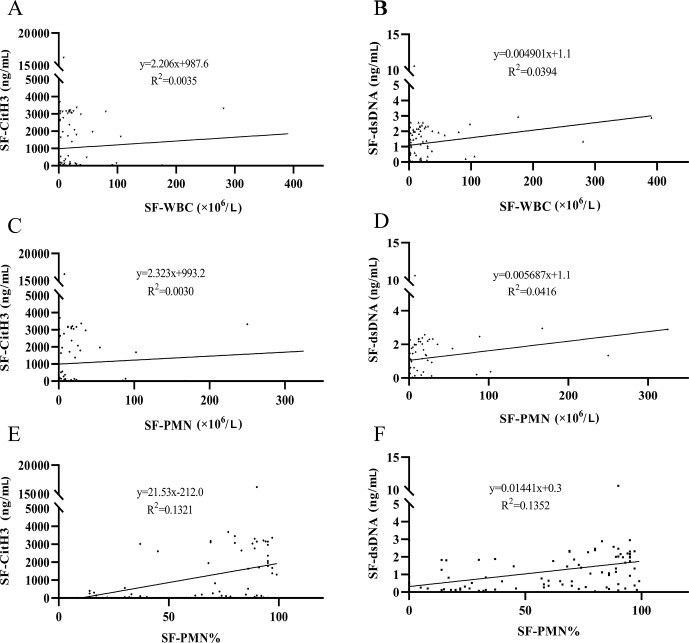
Correlation analysis of SF-CitH3 and SF-dsDNA with SF-WBC, SF-PMN and SF-PMN %, respectively. **(A)** Correlation analysis between SF-CitH3 and SF-WBC. **(B)** Correlation analysis between SF-dsDNA and SF-WBC. **(C)** Correlation analysis between SF-CitH3 and SF-PMN. **(D)** Correlation analysis between SF-dsDNA and SF-PMN. **(E)** Correlation analysis between SF-CitH3 and SF-PMN %. **(F)** Correlation analysis between SF-dsDNA and SF-PMN %. Abbreviations: SF-dsDNA, double-stranded DNA in synovial fluid; SF-CitH3, citrullinated histone H3 in synovial fluid; SF-WBC, white blood cell in synovial fluid; SF-PMN %, polymorphonuclear cell percentage in synovial fluid; SF-PMN, polymorphonuclear cell count in synovial fluid.

A correlation analysis was conducted between NET constituents (SF-dsDNA, SF-Nucleosome and SF-CitH3) and SF-WBC, SF-PMN % and SF-PMN. As shown in Fig. 1, there were positive correlations between SF-dsDNA and SF-WBC ( ρ=0.464 , P < 0.001), SF-PMN % ( ρ=0.455 , P < 0.001) and SF-PMN ( ρ=0.535 , P < 0.001). Similarly, SF-CitH3 also showed significant positive correlations with SF-WBC ( ρ=0.436 , P < 0.001), SF-PMN % ( ρ=0.446 , P < 0.001) and SF-PMN ( ρ=0.494 , P < 0.001) (Fig. 1). SF-Nucleosome was not significantly correlated with local WBC-related indices ( P>0.050 ).

### 3.3 Diagnostic efficacy evaluation for the identified indicators

**Figure 2:**
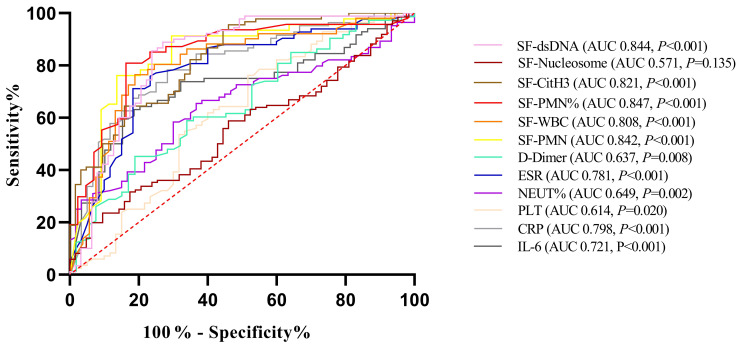
Receiver operating characteristic curve analysis of the identified indicators for periprosthetic joint infection. Abbreviations: SF, synovial fluid; SF-dsDNA, double-stranded DNA in synovial fluid; SF-Nucleosome, nucleosome in synovial fluid; SF-CitH3, citrullinated histone H3 in synovial fluid; SF-PMN %, polymorphonuclear cell percentage in synovial fluid; SF-WBC, white blood cell in synovial fluid; SF-PMN, polymorphonuclear cell count in synovial fluid; ESR, erythrocyte sedimentation rate; NEUT %, neutrophil percentage; PLT, platelet; CRP, C-reactive protein; IL-6, interleukin-6.

ROC analyses were performed for all three components of SF-NETs, as well as for all indices that differed significantly between the PJI and non-PJI groups, including SF-WBC, SF-PMN %, SF-PMN, D-dimer, ESR, NEUT %, PLT, CRP and IL-6. The ROC curves of the different indicators are depicted in Fig. 2. As shown in Table 3, the AUC values of SF-dsDNA (0.844) and SF-CitH3 (0.821) were both greater than 0.800, and the AUC values of SF-PMN % (0.847) and SF-PMN (0.842) were similar to the AUC value of SF-dsDNA. The AUC values of SF-Nucleosome, SF-WBC, D-dimer, ESR, NEUT %, PLT, CRP and IL-6 were 0.571, 0.808, 0.637, 0.781, 0.649, 0.614, 0.798 and 0.721, respectively. The cutoff values of these indicators were calculated by the Youden index as 1.038 ng mL^−1^ (SF-dsDNA), 2.050 (SF-Nucleosome), 1171.694 ng mL^−1^ (SF-CitH3), 2095.000 × 10^6^ L^−1^ (SF-WBC), 68.500 % (SF-PMN %), 947.800 × 10^6^ L^−1^ (SF-PMN), 1.195 mg L^−1^ FEU (D-dimer), 18.500 mm h^−1^ (ESR), 66.900 % (NEUT %), 267.000 × 10^9^ L^−1^ (PLT), 1.235 mg dL^−1^ (CRP) and 8.915 pg mL^−1^ (IL-6). The sensitivity, specificity, PPV and NPV of the identified factors were calculated using their cutoff values, and SF-CitH3 displayed the highest specificity (95.5 %) (Table 3).

**Table 3:** Diagnostic values of the identified indicators.

Parameters	Cutoff value^∗^	Sensitivity %	Specificity %	PPV %	NPV %	AUC	95 % CI	P value
SF-dsDNA, ng mL^−1^	1.038	73.4	89.9	83.9	82.5	0.844	0.776–0.912	< 0.001
SF-Nucleosome	2.050	31.3	88.8	66.7	64.2	0.571	0.477–0.664	0.135
SF-CitH3, ng mL^−1^	1171.694	53.1	95.5	89.5	73.9	0.821	0.756–0.887	< 0.001
SF-WBC, × 10^6^ L^−1^	2095.000	81.1	76.5	78.2	79.6	0.808	0.721–0.895	< 0.001
SF-PMN %	68.500	84.4	80.9	80.9	84.4	0.847	0.763–0.930	< 0.001
SF-PMN, × 10^6^ L^−1^	947.800	86.4	76.1	77.6	85.4	0.842	0.756–0.927	< 0.001
D-dimer, mg L^−1^ FEU	1.195	80.0	45.2	52.4	75.0	0.637	0.541–0.734	0.008
ESR, mm h^−1^	18.500	81.7	71.1	67.1	84.3	0.781	0.702–0.860	< 0.001
NEUT %	66.900	63.3	65.5	56.7	71.4	0.649	0.559–0.738	0.002
PLT, × 10^9^ L^−1^	267.000	48.3	76.2	59.2	67.4	0.614	0.517–0.711	0.020
CRP, mg dL^−1^	1.235	71.7	78.3	70.5	79.3	0.798	0.724–0.873	< 0.001
IL-6, pg mL^−1^	8.915	81.4	63.1	60.8	82.8	0.721	0.637–0.805	< 0.001

^∗^ All cutoff values were determined by the Youden index. Abbreviations: SF, synovial fluid; SF-dsDNA, double-stranded DNA in synovial fluid; SF-Nucleosome, nucleosome in synovial fluid; SF-CitH3, citrullinated histone H3 in synovial fluid; SF-WBC, white blood cell count in synovial fluid; SF-PMN %, polymorphonuclear cell percentage in synovial fluid; SF-PMN, polymorphonuclear cell count in synovial fluid; ESR, erythrocyte sedimentation rate; NEUT %, neutrophil percentage; PLT, platelet; CRP, C-reactive protein; IL-6, interleukin-6; PPV, positive predictive value; NPV, negative predictive value; AUC, area under the curve; CI, confidence interval.

### 3.4 The performance of SF-NET components

To further analyze the diagnostic value of SF-NETs for PJI, different combinations were identified. For instance, according to the ROC curve analysis, SF-NETs_2+_ was categorized when the test values of two out of the three components were equal to or greater than their respective cutoff values. Similarly, SF-NETs_1+_ and SF-NETs_3+_ were also documented (Table 4). As shown in Table 4, SF-NETs_1+_ displayed the greatest sensitivity (82.8 %), and the sensitivity of SF-NETs_2+_ and SF-NETs_3+_ were 59.4 % and 15.6 %, respectively. Meanwhile, SF-NETs_3+_ exhibited the largest specificity of 100.0 %, and the specificity of SF-NETs_1+_ and SF-NETs_2+_ were 78.7 % and 95.5 %, respectively. Additionally, among the NETs_1+_ false-negative subjects, four cases yielded positive cultures, comprising three cases of CoNS and one case of enterococci.

**Table 4:** Combinations of NET components for PJI recognition^∗^.

Items	PJI group, n=64	Non-PJI group, n=89	P value
NETs_1+_, n ( n %)	53 (82.8 %)	19 (21.3 %)	< 0.001
NETs_2+_, n ( n %)	38 (59.4 %)	4 (4.5 %)	< 0.001
NETs_3+_, n ( n %)	10 (15.6 %)	0 (0.0 %)	< 0.001

^∗^ Subjects with one, two and three out of three components of NETs being positive were marked as NETs_1+_, NETs_2+_ and NETs_3+_, respectively. Abbreviations: PJI, periprosthetic joint infection; NETs, neutrophil extracellular traps.

### 3.5 Sifting of risk factors for PJI

A binomial logistic regression analysis was conducted to identify the risk factors of PJI. Variables that were independently and significantly different between the PJI and non-PJI groups – including age, SF-NETs_1+_, SF-PMN %, SF-WBC, Culture, D-dimer, ESR, NEUT %, WBC, PLT, CRP, and IL-6, were included into the analysis. Three indices, namely SF-NETs_1+_, SF-PMN % and Culture – were independent risk factors for PJI (Table 5). A larger number of SF-NETs_1+_ ( P=0.001 ) and Culture ( P=0.010 ) were associated with the increased risk of PJI. Furthermore, high levels of SF-PMN % ( P=0.003 ) increased the risk of PJI.

**Table 5:** Independent risk factors for PJI.

Risk factors	β value	OR value	OR of 95 % CI	P value
NETs ${}_{1+}^{*}$	2.354	10.529	2.615–42.402	0.001
SF-PMN %	3.336	28.114	3.029–260.939	0.003
Culture	2.105	8.210	1.653–40.789	0.010

^∗^ Subjects with one out of three components of NETs being positive were marked as NETs_1+_. Abbreviations: PJI, periprosthetic joint infection; NETs, neutrophil extracellular traps; SF-PMN %, polymorphonuclear cell percentage in synovial fluid fibrinogen; CI, confidence interval; OR, odds ratio.

**Figure 3:**
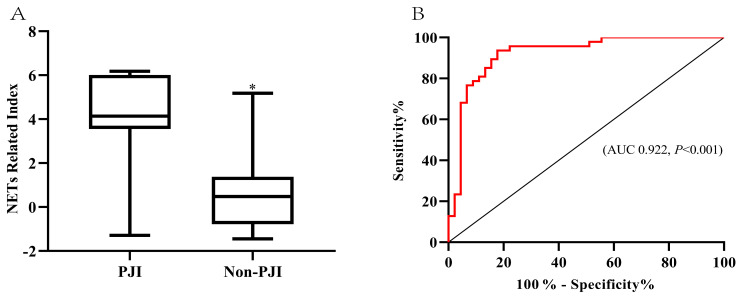
The diagnostic value of NET related index for periprosthetic joint infection. **(A)** Comparison of the NET related index between patients with or without PJI. ^*^ P < 0.050, and the difference is statistically significant. For the box plots shown, the horizontal bars represent medians and the vertical bars show the 2.5th and 97.5th percentiles. **(B)** Receiver operating characteristic curve analysis of NET related index for periprosthetic joint infection. Abbreviations: PJI, periprosthetic joint infection; NET, neutrophil extracellular traps.

Consequently, a formula was developed to calculate a new indicator named NET related index (NETRI): NETRI = 10.529 × SF-NETs ${}_{1+}\;+\;28.114$ × SF-PMN % + 8.210 × Culture - 1.452. In this formula, the positive results of SF-NETs_1+_ and Culture were marked as “1” and their negative results were marked as “0”. As shown in Fig. 3A, compared to patients without PJI, patients with PJI exhibited significantly higher levels of NETRI [0.483 ( - 0.751, 1.376) vs. 4.138 (3.571, 6.009), P < 0.001]. The ROC curve analysis was conducted for NETRI (Fig. 3B), yielding an AUC of 0.922 (95 % CI: 0.862–0.981) with a cutoff value of 3.346. The sensitivity, specificity, PPV and NPV of NETRI were 82.2 %, 93.6 %, 92.5 % and 84.6 %, respectively.

## 4 Discussion

PJI, a severe complication of arthroplasty, has increased in incidence recently. Despite efforts, integrating traditional and novel markers for accurate PJI diagnosis remains challenging (Parvizi et al., 2018). Neutrophils dominate in PJI and release NETs through NETosis. NETs indicate neutrophil activation and may aid PJI diagnosis (Papayannopoulos, 2018). While NETs show value in systemic infections (Kumar et al., 2019), few studies address their role in PJI, and prior works measured NETs incompletely or with small samples (Cai et al., 2023; Lögters et al., 2009; Cobra et al., 2022; de Sandes Kimura et al., 2024). This study, with a larger cohort, measured SF-dsDNA, SF-CitH3 and SF-Nucleosome as NET components. Significant differences were observed in SF-dsDNA and SF-CitH3, but not in SF-Nucleosome, between PJI and non-PJI groups. SF-Nucleosome degradation into dsDNA and histones may explain this (Cutter and Hayes, 2015). Subsequently, we analyzed different numbers of NET components. SF-NETs_1+_ demonstrated higher sensitivity (82.8 %), while SF-NETs_2+_ exhibited higher specificity (95.5 %) with acceptable sensitivity (59.4 %). Given PJI's irreversible damage, sensitive screening with SF-NETs_1+_ suited initial visits; SF-NETs_2+_ better supports specific post-treatment confirmation. This tiered approach may prove useful.

Routine SF-WBC and SF-PMN % performed well for PJI diagnosis (Lee et al., 2017) and was further confirmed in our study, along with SF-PMN (all presented an AUC > 0.800). Therefore, aseptic arthrocentesis remains essential, with confirmed low iatrogenic infection risk (Keating et al., 2023), although such risk accumulates with repeated SF harvest. Furthermore, SF-dsDNA and SF-CitH3 positively correlated with local WBC indicators (0.4 < ρ < 0.7), consistent with NET origins (Dilley et al., 2023).

Pathogen culture, long the “gold standard” for PJI (Lagier et al., 2015), showed only 54.1 % sensitivity with 86.8 % specificity. Such high false negatives necessitated repeated sampling and limited practicability in drug sensitivity assessment and antibiotics selection (Bettencourt and Linder, 2010). There were four positive Culture results observed in NET_1+_ false-negative subjects. This may be attributable to prolonged disease duration, potential suboptimal joint fluid storage and random errors. However, the limited number of culture results and substantial missing data confer a high risk of bias, markedly limiting the reference value. Systemic markers (PLT, ESR, WBC, NEUT %, NEUT count, CRP, D-dimer, IL-6) showed limited diagnostic value (most AUC < 0.800), as circulatory indices poorly reflect local PJI, and were therefore adopted by clinical guidelines as combined markers or with weight points assigned (Sigmund et al., 2022).

Logistic regression identified SF-NETs_1+_, SF-PMN % and Culture as independent risk factors, which were all local indices. A new composite indicator, NETRI, achieved an AUC of 0.922, incorporating and outperforming all of these three markers. SF-NETs_1+_ was included due to its higher sensitivity, which was more suitable for uncertain PJI diagnosis. However, the current NET assay kits involved cumbersome procedures, and simplification of the kits is essential to meet clinical demands.

Indeed, determining the components of NETs separately was still quite complicated for clinical settings. Therefore, it is necessary to design an integrated assay kit for NETs and to conduct necessary validation. A meta-analysis indicated that SF-PMN % has a superior predictive value in PJI, with a sensitivity and specificity of 89 % and 86 % (Lee et al., 2017). Our study also confirmed SF-PMN % as another independent predictor for PJI. Additionally, as the “gold standard”, Culture was also included.

Patients with PJI in our study were 3 years older than those in the non-PJI group, and the median ages were both more than 65 years old. However, age was not identified in the present logistic regression analysis, which aligned with prior studies rejecting age as an independent risk factor (Inoue et al., 2019). While older age affected PJI primarily via other factors rather than independently, it remained noteworthy given the greater comorbidity complexity in elderly patients.

There are several limitations to this study. Our study was a single-center study, which may limit the generalizability of our findings, and future multi-center studies should be conducted. Then, due to missing data, pathogen data were simply described and were not analyzed in detail to avoid bias. Thus, our findings primarily apply to chronic PJI and suggest potential utility of NET-related markers; however, further subgroup analyses by pathogen virulence in larger cohorts are warranted to confirm generalizability, particularly for low-grade organisms. Additionally, the relatively small training set used for developing NETRI might have affected its diagnostic efficacy, and a larger sample size is necessary for validation.

## 5 Conclusions

Two out of the three components of SF-NETs, namely SF-dsDNA and SF-CitH3, were elevated in the PJI group compared to the non-PJI group. SF-dsDNA and SF-CitH3 showed significant positive correlations with local WBC status. SF-NETs_1+_ and SF-NETs_2+_ were proven to be suitable for PJI pre-treatment screening and post-treatment exclusion, respectively. Using binomial logistic analysis, a novel index named NETRI was developed based on SF-NETs_1+_, SF-PMN % and Culture, and can serve as a potential diagnostic indicator for PJI.

## Data Availability

The data that support the findings of this study are available from the corresponding author upon reasonable request. The data cannot be made publicly available due to ethical concerns and privacy restrictions.
